# Aloin Preconditioning Attenuates Hepatic Ischemia/Reperfusion Injury via Inhibiting TLR4/MyD88/NF-*κ*B Signal Pathway *In Vivo* and *In Vitro*

**DOI:** 10.1155/2019/3765898

**Published:** 2019-11-20

**Authors:** Yichao Du, Baolin Qian, Lin Gao, Peng Tan, Hao Chen, Ankang Wang, Tianxiang Zheng, Shilin Pu, Xianming Xia, Wenguang Fu

**Affiliations:** ^1^Academician (Expert) Workstation of Sichuan Province, The Affiliated Hospital of Southwest Medical University, Luzhou 646000, China; ^2^Department of Hepatobiliary Surgery, The Affiliated Hospital of Southwest Medical University, Luzhou 646000, China; ^3^Department of Health Management, The Affiliated Hospital of Southwest Medical University, Luzhou 646000, China; ^4^Nuclear Medicine and Molecular Imaging Key Laboratory of Sichuan Province, Luzhou 646000, China

## Abstract

**Background:**

Aloin exerts considerable protective effects in various disease models, and its effect on hepatic ischemia-reperfusion (HIR) injury remains unknown. This research is aimed at conducting an in-depth investigation of the antioxidant, anti-inflammatory, and antiapoptosis effects of aloin in HIR injury and explain the underlying molecular mechanisms.

**Methods:**

*In vivo*, different concentrations of aloin were intraperitoneally injected 1 h before the establishment of the HIR model in male mice. The hepatic function, pathological status, oxidative stress, and inflammatory and apoptosis markers were measured. *In vitro*, aloin (AL, C_21_H_22_O_9_) or lipopolysaccharide (LPS) was added to a culture of mouse primary hepatocytes before it underwent hypoxia/reoxygenation (H/R), and the apoptosis in the mouse primary hepatocytes was analyzed.

**Results:**

We found that 20 mg/kg was the optimum concentration of aloin for mitigating I/R-induced liver tissue damage, characterized by decreased serum alanine aminotransferase (ALT) and aspartate aminotransferase (AST). Aloin pretreatment substantially suppressed the generation of hepatic malondialdehyde (MDA), tumor necrosis factor alpha (TNF-*α*), and IL-6 and enhanced the hepatic superoxide dismutase (SOD) activities as well as glutathione (GSH) and IL-10 levels in the liver tissue of I/R mice; this indicated that aloin ameliorated I/R-induced liver damage by reducing the oxidative stress and inflammatory response. Moreover, aloin inhibited hepatocyte apoptosis and inflammatory response that was caused by the upregulated expression of Bcl-2, the downregulated expression of cleaved caspase3(C-caspase3), Bax, Toll-like receptor 4 (TLR4), FADD, MyD88, TRAF6, phosphorylated IKK*α*/*β* (p-IKK*α*/*β*), and phosphorylated nuclear factor *κ*B p65 (p-NF-*κ*B p65).

## 1. Introduction

HIR injury is a common clinical practice of organ and tissue injury, which is crucial for liver surgery and patient survival [1]. The hypoxia and nutrient deficiency are causes of initial HIR injury, but inflammatory factors and oxidative stress cause more serious damage to the liver during perfusion; ultimately, it can lead to hepatocyte necrosis and severe tissue damage [2, 3]. Therefore, HIR injury is an important treatment problem that needs to be solved urgently.

TLRs are one of the most representative receptors in innate immune response; Toll-like receptor 4 (TLR4) is capable of recognizing and binding to specific molecular structures of pathogenic microorganisms or host programmed cell death surfaces, such as pathogen-associated molecular patterns and damage-related damage-associated molecular pattern [4]. TLR4 is expressed in monocytes, macrophages, dendritic cells, and parenchymal hepatocytes [5, 6]. To our knowledge, TLR4 signaling includes two classic cascades: myeloid differentiation primary response gene 88- (MyD88-) independent and MyD88-dependent pathways, which can lead to the activation of NF-*κ*B and stimulate the release of proinflammatory cytokines and induces apoptosis [7, 8]. Although more and more studies have shown that activation and inflammatory responses of TLR4 and the mediated signaling pathway play an important role in HIR injury, the mechanism by which innate immune and inflammatory responses mediate HIR injury is not fully clear.

Natural anthraquinone compounds are shown to be safe and effective in hepatic protection. Several researches have indicated that aloin, an anthraquinone stem from the *aloe vera*, and exerted beneficial effects on liver disease [9, 10]. These effects may be attributable, at least in part, to its antioxidative properties. Recent studies have also demonstrated that barbaloin exhibited protective effects on alcohol-induced liver injury in mice via the attenuation of oxidative stress and inflammation. Moreover, AL pretreatment inhibits myocardial oxidative stress via the activation of the AMP-activated protein kinase signaling pathway, thereby alleviating myocardial ischemia-reperfusion injury [10]. The role of AL in immunity, anti-inflammation, and antioxidation has been demonstrated previously [10–12]. However, to our knowledge, no previous study has investigated whether AL pretreatment inhibits hepatocyte apoptosis induced by HIR to achieve hepatocyte protection. Therefore, based on the current knowledge on TLR4/MyD88/NF-*κ*B mediated apoptosis and the hepatic protective effects of AL, the present study is aimed at further evaluating the hepatic protective properties and potential mechanisms of action of AL pretreatment in HIR injury.

## 2. Materials and Methods

### 2.1. Reagents and Animals

Aloin (purity ≥ 97%, Sigma-Aldrich, USA) was dissolved in 0.5% (*w*/*v*) dimethyl sulfoxide (DMSO, Sigma-Aldrich, USA). Lipopolysaccharide (LPS, Solarbio, China) was dissolved in normal saline. All healthy male C57BL/6 mice (8-10 weeks old, weight 18-22 g) were purchased from Chengdu Dashuo Biotechnological Company. All mice were housed in a specific pathogen-free environment (temperature 23°C ± 2°C, humidity 55 ± 5%, and a 12 hr light/dark cycle) with free access to sterile water and food. The study was approved by the Animal Care and Use Committee of Southwest Medical University.

### 2.2. Hepatic I/R Injury Model

Thirty experimental mice were randomly divided into five groups (six per group): Group 1: sham operation control with intraperitoneal injection of normal saline (Sham) for 5 days. Group 2: hepatic ischemia/reperfusion with intraperitoneal injection of normal saline (I/R) for 5 days. Group 3: I/R with intraperitoneal injection of 10 mg/kg AL (I/R+AL10) for 5 days. Group 4: I/R with intraperitoneal injection of 20 mg/kg AL (I/R+AL20) for 5 days. Group 5: I/R with intraperitoneal injection of 40 mg/kg AL (I/R+AL40) for 5 days. A 70% hepatic ischemia model was established according to a previous model described [13]. Mice were anesthetized with sodium pentobarbital (40 mg/kg, i.p.). Its abdominal cavity was opened to expose the hepatic pedicles of the left and middle lobes of the liver. In order to prevent severe mesenteric vein occlusion, the portal vein and hepatic artery of the middle and left lobes were clamped, causing approximately 70% hepatic ischemia. A successful ischemia operation was indicated by the ischemic liver lobes turning white after 0.5 min. The ischemia was maintained for 60 minutes, the clamp was removed for reperfusion then, after reperfusion for 6 h, mice were sacrificed to collect liver samples and serum for subsequent examination.

### 2.3. Liver Function Analyses

The blood of all the mouse were collected and centrifuged for 5 min at 4000 rpm and 4°C. Serum samples were separated and stored at -80°C. Serum aspartate aminotransferase (AST) and alanine aminotransferase (ALT) were measured by colorimetry using a commercially assay kit (Nanjing Jiancheng Bioengineering Institute, China).

### 2.4. Liver Histology and Immunohistochemistry

Liver samples were fixed in 4% paraformaldehyde and embedded in paraffin. Liver histology was observed; hematoxylin and eosin (H&E) and tissue immunohistochemistry (IHC) staining for LY6G (BD Biosciences, China) and TLR4 (Protenintech, China) were performed in paraffin sections according to the specification.

### 2.5. Hepatic GSH, MDA, and SOD Content Assay

Hepatic GSH contents were measured at 405 nm according to ELISA using commercial assay kits (Nanjing Jiancheng Bioengineering Institute, China). Hepatic MDA contents were measured at 532 nm according to thiobarbituric acid colorimetric method using commercial assay kits (Nanjing Jiancheng Bioengineering Institute, China). Hepatic SOD activities were measured at 450 nm by xanthine oxidase method using commercial assay kits (Nanjing Jiancheng Bioengineering Institute, China).

### 2.6. Reactive Oxygen Species (ROS) Production Assay

ROS generation was measured using a ROS Assay Kit (Beyotime, China). In vitro study, primary hepatocytes were incubated with 10 *μ*M DCFH-DA for 30 min at 37°C. Then, the medium was discarded, and cells were washed with ice-cold PBS in the dark, and ROS generation was evaluated by the fluorescence intensity measured also by a fluorescence spectrometry, and images were obtained with a fluorescence microscope (Olympus, Japan).

### 2.7. Tunel Staining

The 5-micron thick paraffin sections were deparaffinized and dehydrated. The apoptosis of the liver tissue was then detected with a TUNEL kit (Beyotime, China) according to the specification.

### 2.8. Detection of Caspase 3 Activity

Liver tissues were added to the cell lysate and homogenized. The tissue was homogenated and centrifuged, and the supernatant was gotten. The caspase 3 activity of liver tissue was detected with a Caspase 3 Activity Assay Kit (Beyotime, China) immediately.

### 2.9. Annexin V-FITC Cell Apoptosis Assay

The primary hepatocyte early apoptosis ratios were analyzed by flow cytometry with Annexin V-FITC/PI Cell Apoptosis Detection Kit (TransGen Biotech, China). In brief, primary hepatocytes were collected and washed two times with precooled PBS, resuspended in 100 *μ*L of precooled 1× Annexin V binding buffer, and added to 5 *μ*L Annexin V-FITC and 10 *μ*L PI for 15 min at room temperature in the dark. Addition of sugar and detection by flow cytometry were made in an hour.

### 2.10. Western Blotting

Western blot was performed as previously described [14]. The following primary antibodies have been used: caspase 3 (1 : 1000), NF-*κ*B p65 (1 : 1000), Bax (1 : 1000), IL-10 (1 : 1000), IL-6 (1 : 1000), TNF-*α* (1 : 1000), FADD (1 : 1000), Bcl-2 (1 : 1000), TLR4 (1 : 1000), MyD88 (1 : 2000), and TRAF6 (1 : 2000), all from Proteintech, China); p-IKK*α*/*β* (1 : 1000, Zen Bioscience, China); and p-NF-*κ*B p65 (1 : 1000, Cell Signaling Technology, China). The membranes were subsequently washed with TBST and incubated for 1 h with horseradish peroxidase- (HRP-) conjugated secondary antibodies. The membranes were detected with BeyoECL Moon (Beyotime, China). Image J software (NIH, USA) was used to detect the grayscale value of straps.

### 2.11. Cell Culture and H/R Model of Primary Hepatocytes

Primary hepatocytes were isolated from male mice aged 8-10 weeks using the collagenase perfusion method as described in previous studies [15]. Primary hepatocytes were cultured in DMEM supplemented with 1% penicillin-streptomycin and 10% fetal bovine serum in plates coated with rat tail collagen at 37°C incubator chamber (Thermo Scientific, USA) with 5% CO_2_. After attachment for 4 hours, the culture medium was changed to sugar-free and serum-free DMEM (HyClone, USA) balanced with 1% O_2_, 5% CO_2_, and 94% N_2_. After 4 hours, cells were incubated under standard culture conditions of 5% CO_2_ in air at 37°C for the indicated times. The cells were divided into five treatment groups. These were control, H/R, 20 *μ*M AL added at the beginning of H/R treatment (H/R+AL), 0.5 *μ*g/ml LPS (LPS) and AL plus 0.5 *μ*g/ml LPS, and a TLR4 activator [16] added 1 h before the addition of AL at the beginning of H/R treatment (H/R+AL+LPS).

### 2.12. RNA Isolation and Real-Time PCR

The procedure was carried out as previously described [17]. The mRNA levels were measured by quantitative real-time RT-PCR (qRT-PCR) analysis based on PerfectStart Green qPCR SuperMix (TransGen, China) with the LightCycler 96 System (Roche, Switzerland). Primers for real-time PCR were as follows from Sangon Biotech (China):

IL-6 forward 5′-TTCTCTGGGAAATCGTGGAAA-3′,

IL-6 reverse 5′-TGCAAGTGCATCATCGTTGT-3′;

IL-10 forward 5′-GCTCTTACTGACTGGCATGAG-3′,

IL-10 reverse 5′-CGCAGCTCTAGGAGCATGTG-3′;

TNF-*α* forward 5′-CCAGTGTGGGAAGCTGTCTT-3′,

TNF-*α* reverse 5′-AAGCAAAAGAGGAGGCAACA-3′;

GAPDH forward 5′-AGGTCGGTGTGAACGGATTTG-3′,

GAPDH reverse 5′-TGTAGACCATGTAGTTGAGGTCA-3′.

Results were normalized to GAPDH.

### 2.13. RNA Interference

Primary hepatocytes were transfected with double-stranded siRNA or negative control siRNA (non-siRNA) using RFect transfection reagent (Bio-Tran Biotechnologies, China). Target sequences were as follows: sense 5′-GAAAUGAGCUG GUAAAGAATT-3′, antisense 5′-UUCUUUACCAGCUCAUUUCTT-3′ for TLR4 [18]. Double-stranded siRNAs were synthesized by Shanghai GenePharma (China).

### 2.14. Statistical Analysis

Statistical analysis was performed using GraphPad Prism 6.0 (GraphPad Software, CA). Differences between experimental groups were compared with one-way ANOVA with Tukey test. Data were expressed as mean ± SD. All differences were considered statistically significant at *P* < 0.05.

## 3. Results

### 3.1. AL-Mitigated I/R-Induced Liver Tissue Damage

**Figure 1:**
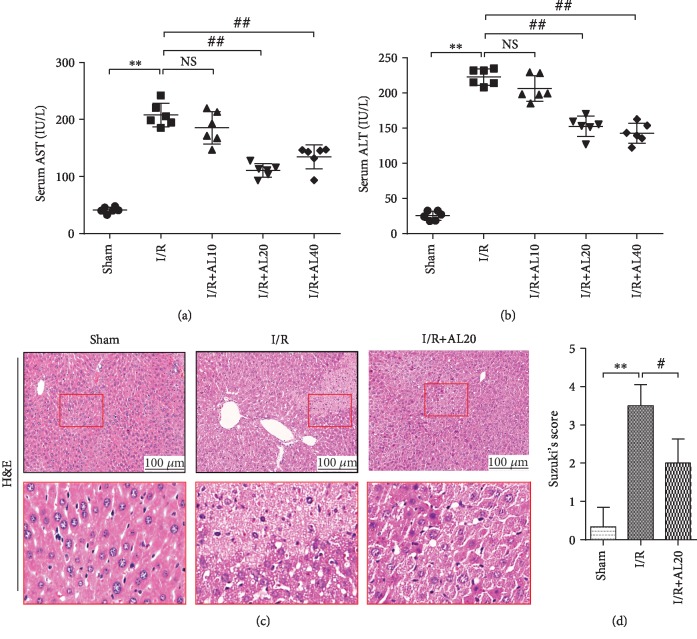
AL decreased liver injury induced by I/R. Serum AST (a) and ALT (b) were assayed after liver ischemia and 6 h of reperfusion with or without intraperitoneal injection of AL. (c) Representative H&E- (original magnification ×200) stained liver sections from sham, I/R, and I/R+AL 20 groups. (d) Histological grading of liver I/R is determined by Suzuki's score. Values represent mean ± standard deviation (SD) values (*n* = 6). ^∗^*P* < 0.05, ^∗∗^*P* < 0.01 versus the sham group; ^#^*P* < 0.05, ^##^*P* < 0.01 versus the I/R group; NS: no significance; one-way ANOVA with Tukey test.

First, we treated the I/R groups with different concentrations of AL and assessed the liver function based on the ALT and AST levels. The serum levels of ALT and AST indicated that the decline in the I/R+AL 20 mg/kg group is the most significant in different concentrations of AL-treated groups when compared with the I/R group (Figures 1(a)and 1(b)) (*P* < 0.01). Finally, we selected 20 mg/kg to be the optimum concentration, as indicated by histologic observation. The sham group exhibited normal morphology; however, the I/R group displayed severe damage and collapses in the hepatic lobular structure, karyopyknosis, inflammatory cell infiltration, and dilatation and congestion hepatic sinus. However, the I/R+AL 20 group had mild degeneration, and hepatocyte nuclei and hepatic cords basically maintained their normal morphology (Figures 1(c) and 1(d)).

### 3.2. AL Suppresses I/R-Induced Liver Tissue Oxidative Stress

**Figure 2:**
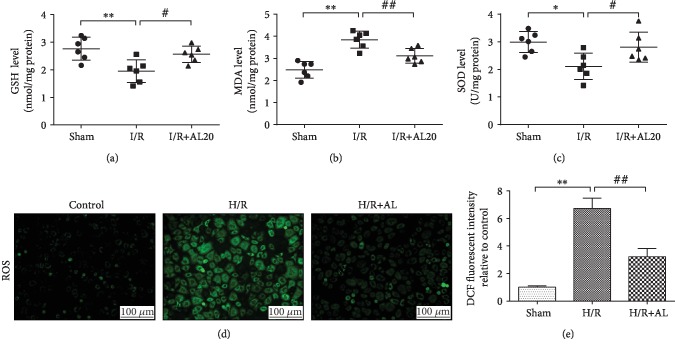
Protective effect of AL against H/R injury through ROS reduction. The hepatic tissue GSH (a) concentration, MDA (b) concentration and SOD (c) activity after 6 h reperfusion. (d, e) Cellular ROS estimated using the probe DCFH-DA by fluorescence microscopy. Values represent mean ± standard deviation (SD) values (*n* = 6). ^∗^*P* < 0.05, ^∗∗^*P* < 0.01 versus the sham (Control) group; ^#^*P* < 0.05, ^##^*P* < 0.01 versus the I(H)/R group; one-way ANOVA with Tukey test.

Intervention effects of AL on I/R-induced liver tissue oxidative stress are shown in Figure 2. The hepatic tissue GSH concentration in the I/R group was significantly lower than that in the sham group; however, the hepatic tissue GSH concentration in the I/R+AL group was significantly higher than that in the I/R group (*P* < 0.05) (Figure 2(a)). The concentration of hepatic tissue MDA, a marker of lipid peroxidation, was significantly higher than that in the sham group, while that in the I/R+AL 20 group was significantly lower than that in the I/R group (*P* < 0.01) (Figure 2(b)). The liver tissue SOD activity in the I/R group was significantly lower than that in the sham group, while that in the I/R+AL 20 group was significantly higher than that in the I/R group (*P* < 0.05) (Figure 2(c)). Furthermore, post H/R oxidative stress in primary mouse hepatocytes by pretreatment with AL manifested as a decrease of DCFH-DA fluorescence than that in the H/R group (*P* < 0.05) (Figures 2(d) and 2(e)). These results indicated that AL suppresses liver tissue and hepatocyte oxidative stress during I/R or hepatocyte H/R injury.

### 3.3. AL Attenuated Inflammatory Response in I/R-Stressed Liver

**Figure 3:**
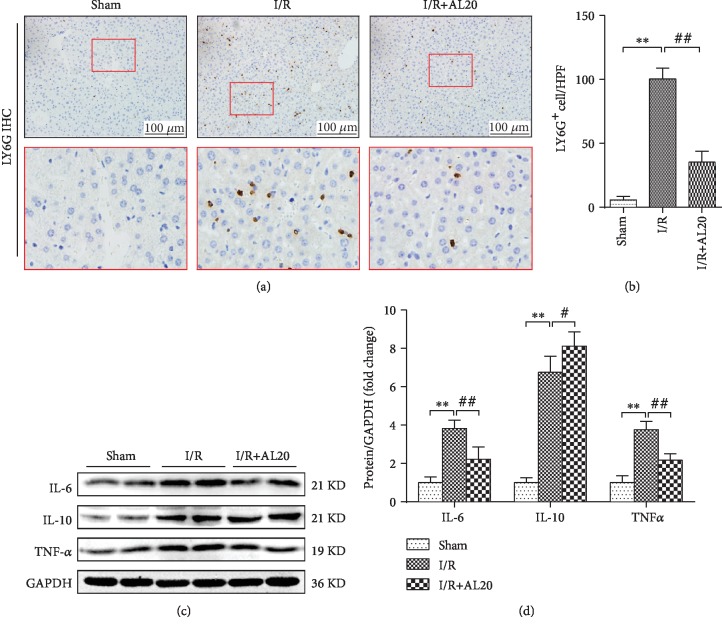
AL attenuated the inflammatory response in I/R-stressed liver. (a, b) Immunohistochemistry analysis of LY6G (original magnification ×200). (c, d) Western blot-assisted analysis of IL-6, IL-10, TNF-*α*, and GADPH. Values represent mean ± standard deviation (SD) values (*n* = 6/group). ^∗^*P* < 0.05, ^∗∗^*P* < 0.01 versus the sham group; ^#^*P* < 0.05, ^##^*P* < 0.01 versus the I/R group; one-way ANOVA with Tukey test.

In order to study the effect of AL on the inflammatory response *in vitro*, inflammatory markers were measured. Figures 3(a) and 3(b) illustrate that the number of infiltrating neutrophils was increased in the I/R tissues; however, the AL-treated group showed less neutrophil accumulation detected using Ly6G-stained immunohistochemistry. To further demonstrate whether AL pretreatment was able to decrease inflammatory response, qPCR and Western blot analysis showed a significant increase in the level of mRNA and protein of IL-6 and TNF-*α*, while that of IL-10 was decreased in the I/R group as compared to that in the sham group. In contrast, AL pretreatment significantly reduced IL-6 and TNF-*α* mRNA and protein levels while it increased that of IL-10 compared with that in the I/R group (Figures 3(c) and 3(d), Supplementary [Supplementary-material supplementary-material-1]).

### 3.4. AL Decreased Hepatocellular Apoptosis after Liver I/R

**Figure 4:**
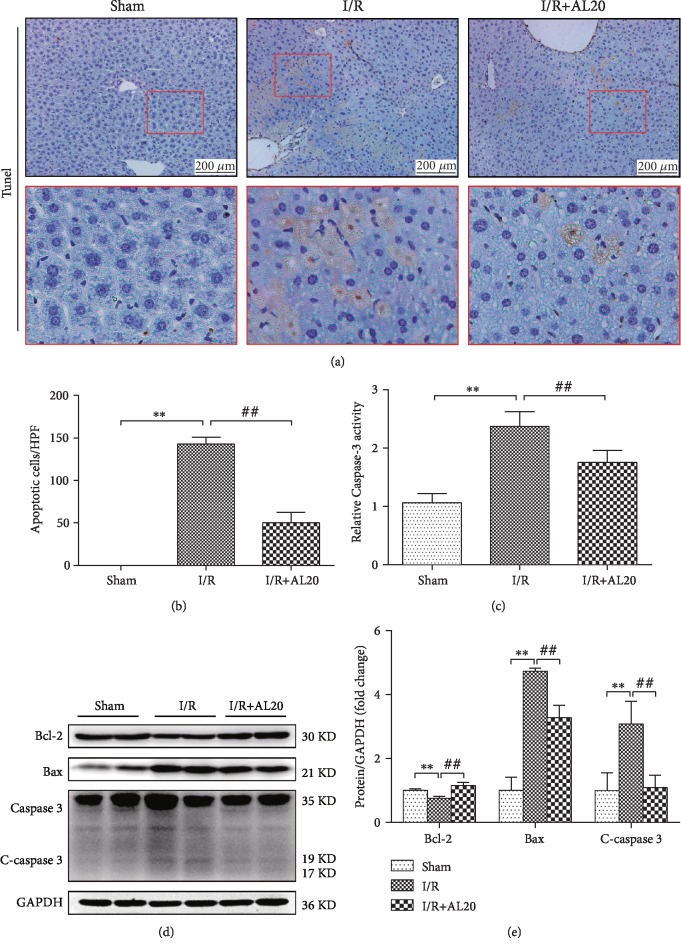
AL decreased hepatocellular apoptosis in IR-stressed liver. (a, b) Representative images of TUNEL (original magnification ×200). (c) Caspase3 activity. (d, e) Western blot-assisted analysis of Bcl-2, Bax, C-caspase 3, and GADPH. Values represent mean ± standard deviation (SD) values (*n* = 6). ^∗^*P* < 0.05, ^∗∗^*P* < 0.01 versus the sham group; ^#^*P* < 0.05, ^##^*P* < 0.01 versus the I/R group; one-way ANOVA with Tukey test.

Apoptosis, a vital progress of cell death, was evaluated using TUNEL. As shown in Figure 4(a), the number of TUNEL-positive hepatocytes in I/R-performed liver was dramatically increased as compared to that in the sham group, attenuated by AL pretreatment. Caspase 3 plays an essential role in cell apoptosis; therefore, the caspase 3 activity was measured. Consistent with the results of TUNEL, the caspase 3 activity was elevated in the I/R group up to 2.2-fold compared with the sham group, and pretreatment mice with AL significantly decreased I/R-induced activity of caspase 3 by approximately 26.3% (Figure 4(c)). We also assayed hepatocellular apoptosis in Western blot that showed that the expression of apoptosis-related proteins was affected by I/R and that AL exerted an antiapoptotic effect (Figures 4(d) and 4(e)).

### 3.5. AL Inhibits the TLR4/MyD88/NF-*κ*B Signal Pathway In Vivo

**Figure 5:**
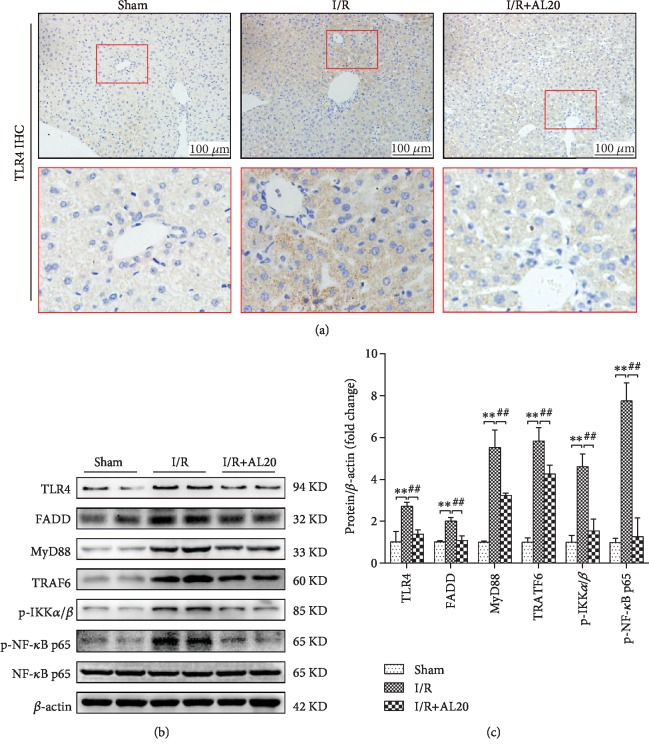
AL inhibits the TLR4/MyD88/NF-*κ*B signal pathway. (a) Immunohistochemistry analysis of TLR4 (original magnification ×200). (b, c) Western blot-assisted analysis of TLR4, FADD, MyD88, TRAF6, p-IKK*α*/*β*, p-NF-*κ*B p65, and *β*-actin. Values represent mean ± standard deviation (SD) values (*n* = 6). ^∗^*P* < 0.05, ^∗∗^*P* < 0.01 versus the sham group; ^#^*P* < 0.05, ^##^*P* < 0.01 versus the I/R group; NS: no significance; one-way ANOVA with Tukey test.

As shown in Figure 5(a), the frequency of TLR4-positive cells was significantly decreased in the IR group as compared to that in the IR+AL 20 group. As per Western blot analysis, liver IR significantly induced TLR4, FADD, and MyD88 expression and phosphorylation of NF-*κ*B p65 and IKK*α*/*β* that was inhibited by AL (Figures 5(b) and 5(c)). These data indicated that AL inhibited the inflammatory responses during liver IR via suppression of the TLR4/MyD88/NF-*κ*B signaling pathway.

### 3.6. AL Inhibited H/R-Induced Apoptosis of Hepatocytes and the TLR4/MyD88/NF-*κ*B Signal Pathway In Vitro

**Figure 6:**
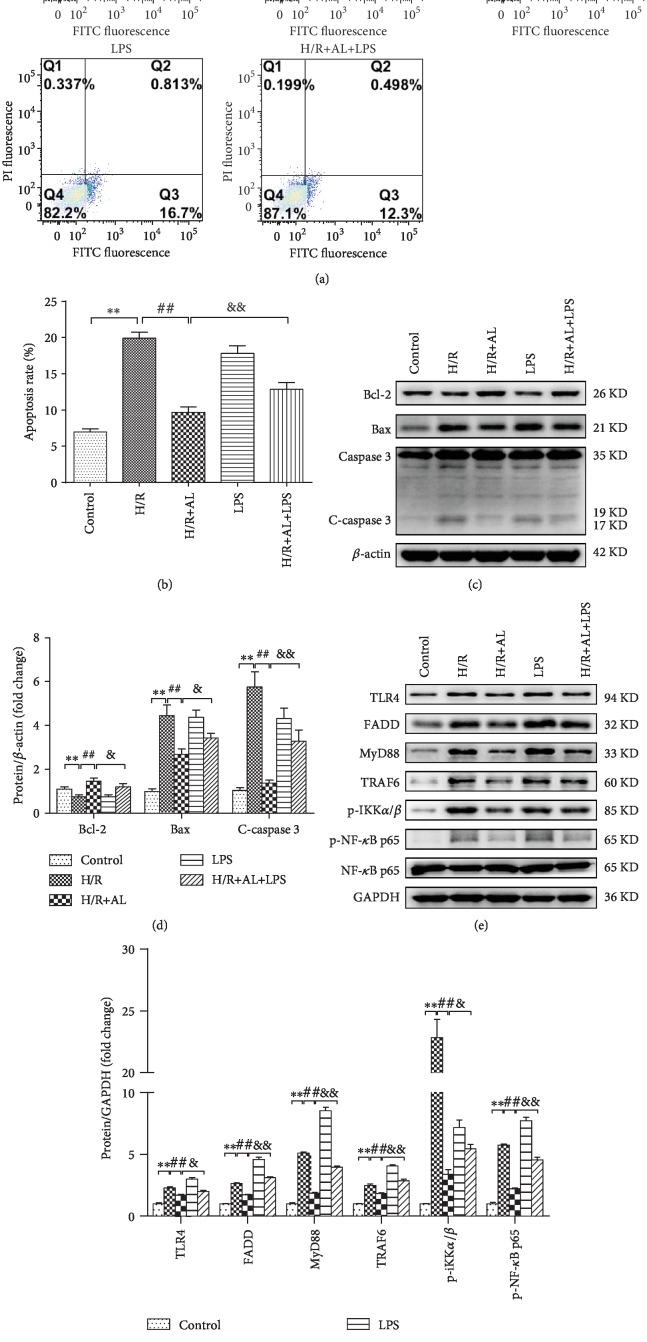
AL inhibits apoptosis of hepatocytes and TLR4/MyD88/NF-*κ*B signal pathway. (a, b) Apoptosis of primary mouse hepatocytes was assayed using flow cytometry. (c, d) Western blot-assisted analysis of Bcl-2, Bax, C-caspase 3, and *β*-actin. (e, f) Western blot-assisted analysis of TLR4, FADD, MyD88, TRAF6, p-IKK*α*/*β*, p-NF-*κ*B p65 and GAPDH. Values represent mean ± standard deviation (SD) values (*n* = 6). ^∗^*P* < 0.05, ^∗∗^*P* < 0.01 versus the control group; ^#^*P* < 0.05, ^##^*P* < 0.01 versus the H/R group; ^&^*P* < 0.05, ^&&^*P* < 0.01 versus the H/R+AL group; one-way ANOVA with Tukey test.

To further understand whether apoptosis occurred in primary mouse hepatocytes, flow cytometry was used to analyze the apoptosis rate. As shown in Figures 6(a) and 6(b), the hepatocyte apoptosis percentage of the control group was 6.99% ± 0.40%, the hepatocyte apoptosis rate of the H/R group was 19.90% ± 0.87%, the hepatocyte apoptosis rate of the H/R+AL group was 9.66% ± 0.88%, the hepatocyte apoptosis rate of the LPS group was 17.79% ± 1.09%, and the hepatocyte apoptosis rate of the H/R+AL+LPS group was 12.85% ± 0.94%. The percentage of apoptotic cells increased with H/R treatment. AL decreased the apoptosis percentage, but the effect of AL was decreased by LPS. It was suggested that AL decreased H/R-induced apoptosis of primary mouse hepatocytes. According to Western blot analysis, hepatocyte H/R significantly induced Bax, C-caspase3, TLR4, FADD, MyD88, TRAF6 expression, and p-NF-*κ*B p65, p-IKK*α*/*β*, which was inhibited by AL. LPS significantly induced Bax, c-caspase3, TLR4, FADD, MyD88, TRAF6, p-iKK*α*/*β*, and p-NF-*κ*B p65 expression, which was inhibited by AL.(Figures 6(c)–(f)). These data indicated that AL inhibits apoptosis of primary mouse hepatocytes during primary mouse hepatocyte H/R via suppressing the TLR4/MyD88/NF-*κ*B signaling pathway.

### 3.7. TLR4 Deficiency Attenuates H/R-Induced Hepatocyte Apoptosis and Downstream Molecule Activation

**Figure 7:**
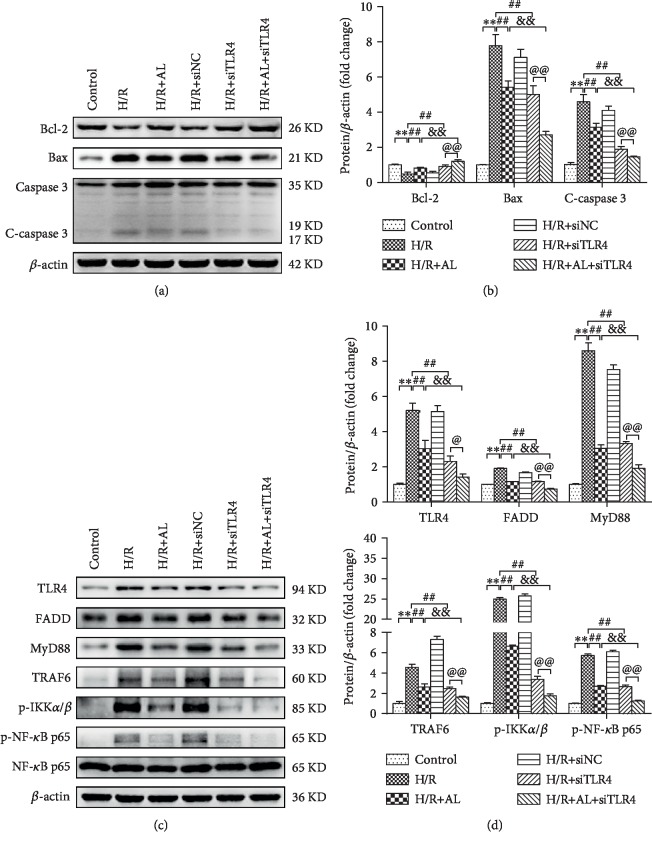
TLR4 deficiency attenuates H/R-induced hepatocyte apoptosis and downstream molecule activation. (a, b) Western blot-assisted analysis of Bcl-2, Bax, C-caspase 3, and *β*-actin. (c, d) Western blot-assisted analysis of TLR4, FADD, MyD88, TRAF6, p-IKK*α/β*, p-NF-*κ*B p65, NF-*κ*B p65, and GAPDH. Values represent mean ± standard deviation (SD) values (*n* = 6). ^∗^*P* < 0.05, ^∗∗^*P* < 0.01 versus the control group; ^#^*P* < 0.05, ^##^*P* < 0.01 versus the H/R group; ^&^*P* < 0.05, ^&&^*P* < 0.01 versus the H/R+AL group;^@^*P* < 0.05, ^@@^*P* < 0.01 versus the H/R+siTLR4 group; one-way ANOVA with Tukey test.

To better explain that AL mainly regulate the TLR4/MyD88/NF-*κ*B pathway for hepatocyte protection against H/R injury, TLR4 was downregulated by siRNA in mouse hepatocytes for 48 h. As shown in Figures 7(a)–7(d), we observed that there was significant difference between the control group and the H/R group (*P* < 0.01). The protein levels of Bax, C-caspase3, TLR4, FADD, MyD88, TRAF6, p-IKK*α*/*β*, and p-NF-*κ*B p65 in the H/R+siTLR4 group were significantly decreased, and antiapoptotic Bcl-2 proteins were significantly increased compared with the H/R group (*P* < 0.01), which means TLR4 deficiency attenuates H/R-induced hepatocyte apoptosis and downstream molecule activation. The protein levels of Bax, C-caspase3, TLR4, FADD, MyD88, TRAF6, p-IKK*α*/*β*, and p-NF-*κ*B p65 in the H/R+AL+siTLR4 group were significantly decreased compared with those in the H/R+AL group (*P* < 0.05), which shows silencing of TLR4 enhanced the beneficial effects of AL.

## 4. Discussion

HIR injury is a major reason for postoperative hepatic dysfunction and liver failure after liver resection, liver transplantation, and trauma surgery [19]. Therefore, the prophylaxis and treatment of HIR injury have become one of the challenges and an important topic of clinical research. It is now thought that the most promising methods for anti-HIR injury in the future are pretreatment and pharmacological approaches [20]. More and more researchers have been investigating the protective effects of herbal medicines during HIR injury for the past few years [21–23].

Aloe vera is a historically renowned topical treatment for abrasions and burns as well as an emollient and moisturizer used in the cosmetics industry [24]. AL is one of the main active components in the leaf exudates of the *Aloe vera* plant, accounting for about 15%–40% [25]. An extensive literature review indicates that AL significantly increases the liver enzymes and improves the pathological changes in liver tissues in the models of chronic alcoholic liver injury [26] and thioacetamide- (TAA-) induced hepatic retinopathy [27]. Moreover, the protective effect of AL was documented before in I/R injury that occurred in the PC12 cells [28] and the heart [10, 29]. Our study first demonstrated that AL could protect the hepatic function in I/R mice and is related to the inhibition of oxidative stress, inflammation response, and apoptosis, mainly via the regulation of the TLR4/MyD88/NF-*κ*B pathway, a potential mechanism underlying its effect.

Currently, it is generally believed that the degree of injury in the reperfusion period is more serious than that in ischemia because a large amount of ROS is produced in the reperfusion process [30]. These ROS can directly damage the liver tissues and lead to apoptosis, vascular dilation, and organ failure [10, 31]. Hence, there is no doubt about ROS reduction during HIR [32]. Many previous researches have indicated that AL reduced the levels of intracellular ROS and increased the antioxidant activity [26, 33, 34]. AL suppressed the generation of hepatic MDA and enhanced the hepatic SOD activities and the level of GSH in the I/R liver in this research. Studies also prove that AL suppresses liver tissue and hepatocyte oxidative stress during I/R or hepatocyte H/R injury (Figure 2). These results show that the prevention of HIR injury by AL is partially attributable to its oxidation resistance.

Furthermore, HIR injury results in an intricate release of inflammatory factors and the activation of multiple inflammatory cascades [35]. TNF-*α* is a well-known mediator during sterile inflammation in HIR injury that plays a central role in liver injury [36]. In the present study, AL reduced the number of infiltrating neutrophils as well as TNF-*α* and IL-6 levels while increasing IL-10 during HIR injury, in keeping with previous reports [11, 37]. However, TNF-*α* also induces the recruitment of neutrophils via the expression of adhesion molecules and stimulates chemokines that lead to the recruitment of neutrophils resulting in the release of more ROS and proteases, creating further injury [38]. In contrast, ROS also triggers the release of cytokines in the liver, heart, and kidneys [39]. Our findings are in accordance with other studies in which cytokine release increased significantly during HIR injury (Figure 3(c)).

Owing to the universal expression of death receptor in the organs, hepatocytes and cholangiocytes are particularly susceptible to death receptor-mediated apoptosis [40]. TLR4 is widely expressed in human cells as a DR that needs to combine with FADD to induce apoptosis [41]. In our research, we provide evidence that AL was able to suppress apoptosis in both in vitro and in vivo experiments. The results showed that AL exaggerated apoptosis by downregulating TLR4, FADD, and C-caspase3 expression. Our study provides a novel link between TLR4-FADD-Caspase3 signaling and AL against apoptosis in HIR or hepatocyte H/R injury (Figures 4(d), 5(b), and 6). In addition, the Bcl-2 protein family is also an important protein that regulates apoptosis, has an obvious antiapoptotic effect, promotes the expression of bcl-2 protein, and alleviates tissue damage caused by HIR injury. Our results showed that AL downregulated the Bax expression and upregulated Bcl-2 in HIR injury, providing further evidence that AL decreased hepatocellular apoptosis after HIR (Figure 4(d)).

In addition, activated TLR4 induced innate immune response independent of the MyD88 (myeloid differentiation factor) signaling pathway and activated NF-*κ*B signaling pathways to induce a series of gene activation and inflammatory cytokine secretion including TNF-*α*, leading to systemic inflammatory response [42]. In the animal experiment, our results revealed that the protein expression of hepatic TLR4, MyD88, p-IKK*α*/*β* and p-NF-*κ*B p65 was markedly decreased in the I/R+AL group as compared with that in the I/R group (Figure 5(b)), consistent with previous reports [43, 44]. Then, we used synthetic TLR4-siRNA to knockdown TLR4 expression. When TLR4 was knocked down in the H/R model of primary hepatocytes, the protein expression levels of TLR4, MyD88, TRAF6, p-IKK*α/β*, and p-NF-*κ*B p65 were also inhibited by AL (Figure 7(c)). Moreover, AL reduces inflammatory gene iNOS via the inhibition of the activation of NF-*κ*B in a model of LPS-induced lung injury [34, 45]. Luo confirmed that AL suppresses LPS-induced inflammatory response and apoptosis by inhibiting the activation of NF-*κ*B in RAW 264.7 cells [11].

## 5. Conclusion

In this study, we provided adequate evidence that AL is an effective protective agent in HIR injury *in vivo* and *in vitro*, mainly through its antioxidative, anti-inflammatory, and antiapoptosis properties. Moreover, we found that AL mainly regulated the TLR4/MyD88/NF-*κ*B pathway for liver protection against HIR injury. Therefore, AL may be a potential candidate for the adjuvant therapy.

## Data Availability

Data used to support the findings of this study are available upon request.

## Supplementary Materials

Supplementary Materials Supplementary Figure 1: (A-C) cytokine gene (IL-6, IL-10, and TNF-*α*) expression in livers harvested 6 hours after reperfusion by quantitative RT-PCR analysis. Supplementary Figure 2: expression of TLR4 in primary mouse hepatocytes transfected with siTLR4 detected through Western blotting.
