# Supplementary material for: Aloin Preconditioning Attenuates Hepatic Ischemia/Reperfusion Injury via Inhibiting TLR4/MyD88/NF-κB Signal Pathway In Vivo and In Vitro
Source: Oxid Med Cell Longev. 2019 Nov 20;2019:3765898. doi: 10.1155/2019/3765898 (PMC6886335; doi:10.1155/2019/3765898)
Supplement: Supplementary Materials — Supplementary Figure 1: (A-C) cytokine gene (IL-6, IL-10, and TNF-α) expression in livers harvested 6 hours after reperfusion by quantitative RT-PCR analysis. Supplementary Figure 2: expression of TLR4 in primary mouse hepatocytes transfected with siTLR4 detected through Western blotting. [file 3765898.f1.pdf]

Aloin Preconditioning Attenuates Hepatic Ischemia/Reperfusion Injury via inhibiting TLR4/MyD88/NF-κB Signal Pathway *in Vivo* and *in Vitro*

Yichao Du [1,2], Baolin Qian [2], Lin Gao [3], Peng Tan [1,2], Hao Chen [2], Ankang Wang [2], Tianxiang Zheng [1,2], Shilin Pu [2], Xianming Xia [1,2], Wenguang Fu [1,2,4]

[1]*Academician (Expert) Workstation of Sichuan Province, the Affiliated Hospital of Southwest Medical University, Luzhou 646000, China*

[2]*Department of Hepatobiliary Surgery, the Affiliated Hospital of Southwest Medical University, Luzhou 646000, China*

[3]*Department of Health Management, the Affiliated Hospital of Southwest Medical University, Luzhou, 646000, China*

[4]*Nuclear Medicine and Molecular Imaging Key Laboratory of Sichuan Province, Luzhou 646000, China*

Correspondence should be addressed to Wenguang Fu; fuwg@swmu.edu.cn

Supplementary results

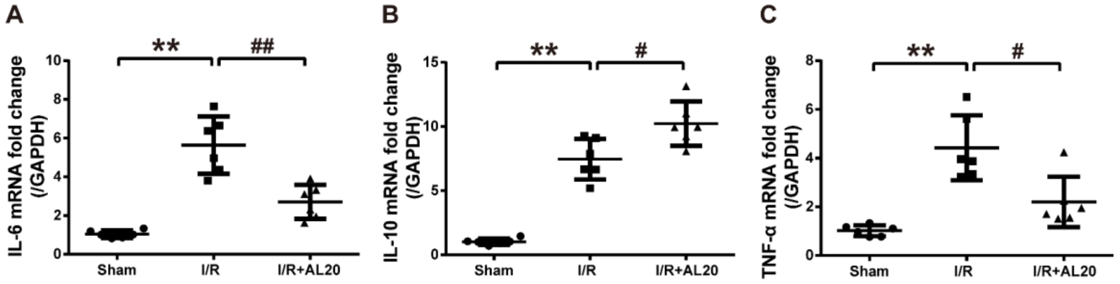

Supplementary Figure 1 AL regulates inflammatory response in IR-stressed liver. (A-C) Cytokine gene (IL-6, IL-10 and TNF-α) expression in livers harvested 6 hours after reperfusion by Quantitative RT-PCR analysis. Values represent mean ± standard deviation (SD) values (n = 6). *$P < 0.05$, **$P < 0.01$ versus the Sham group; $^{\#}P < 0.05$, $^{\#\#}P < 0.01$ versus the I/R group; one-way ANOVA with Tukey test.

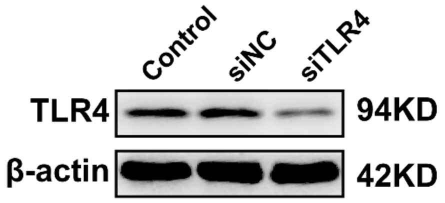

Supplementary Figure 2 Expression of TLR4 in primary mouse hepatocytes transfected with siTLR4 detected through western blotting.
